# Injuries and Strength Training Practices in Collegiate Tennis

**DOI:** 10.3390/sports10100149

**Published:** 2022-09-29

**Authors:** Ecaterina Vasenina, William B. Hammert, Ryo Kataoka, Scott J. Dankel, Samuel L. Buckner

**Affiliations:** 1 Exercise Science Program, University of South Florida, Tampa, FL 33620, USA; 2 Department of Health and Exercise Science, Rowan University, Glassboro, NJ 08028, USA

**Keywords:** strength and conditioning, college tennis, NCAA, injury prevention

## Abstract

Strength and conditioning practices may influence injury rates in the sport of tennis. Methods: Coaches reported the number injuries over the past year. Coaches were also surveyed on whether their training program included training related to upper-body or lower-body strength, power, muscle growth, and eccentric exercise. Separate regression analyses were run in the upper and lower body to examine the relationship between injuries and participation in training focused on strength, power, growth, and maximal eccentric exercise. A total of 111 coaches were surveyed. The most frequent injuries observed were ankle sprains (144 injures), followed by paraspinal muscle strains (126 injuries). When pooled, there were a total of 355 lower-body and 260 upper-body injuries. Strength and conditioning practices explained 9.9% of the variance of injury rates in the upper body (R^2^ = 0.099). The only significant predictor of upper-body injury was participation in upper-body muscle growth training (β = 1.613, *p* = 0.013). In addition, training practices explained 11.1% of the variance of injury in the lower body (R^2^ = 0.111). Coaches value injury prevention exercise, sports-specific training and flexibility and mobility training the most, with muscle growth and maximal power ranked lowest. Additionally, the most frequent injuries observed in collegiate tennis players were ankle sprains (144 injures), followed by paraspinal muscle strains (126 injuries).

## 1. Introduction

Tennis is a physically demanding sport that requires a high level of conditioning in order to facilitate injury prevention [1]. Extensive repetitive motion with high-velocity arm movements may result in overuse injuries in the upper extremities, while sprinting to the tennis ball, stopping, and pounding place a strain on lower extremities and might increase the risk of acute and/or overuse injuries [2]. Safran et al. [3] conducted a questionnaire assessment during the Junior National Tennis Championship in 1998 and discovered that 23% of female tennis players and 43% of male tennis players were injury-free, with more than 53% of females and 29% of males reporting occurrence of at least one injury during their tennis career [3]. In addition, previous studies have reported that the majority of injuries in collegiate athletes are diagnosed as strains (men: 30.9%; women: 29.1%), sprains (men: 14.4%; women: 15%), inflammation (men: 10.5%; women: 10.1%), and tendonitis (men: 6.1%; women: 7.9%) [4]. The most prevalent injuries include lateral ankle ligament complex sprains, trunk injuries (strains), and shoulder/clavicle injuries [4].

The injuries experienced in the sport of tennis may (in part) be a result of the significant musculoskeletal stress of the sport. Some insight is provided by Ojala and Häkkinen [5] whom examined changes in physiological and performance variables in male tennis players following a 3-day long tennis tournament. Eight tennis players (age 23.0 ± 3.8) played three 2 h long tennis matches followed by a 2-day long recovery period. Following completion of the study, levels of creatine kinase were elevated, peaking following the third match, and remaining elevated above baseline following one day of rest. Upper- and lower-body delayed-onset muscle soreness (DOMS) was elevated throughout the tournament and, similarly to creatine kinase, remained elevated following one day of rest. Additionally, both maximal voluntary contraction (MVC) and rate of force development (RFD) decreased throughout the tournament, with no difference between players who won and players who lost their matches, indicating that merely participating in the tennis tournament (not necessarily advancing) may induce a significant musculoskeletal stress. This is further supported by the findings of Gescheit et al. [6], who observed increased levels of creatine kinase, ratings of muscle soreness, and fatigue (observed through countermovement jump height and isometric mid-thigh pull peak force reduction) following 4 h long tennis matches that were played on 4 consecutive days. Both abovementioned studies demonstrate that 3–4 days of consecutive tennis matches might cause increased soreness, reduced recovery, and elevated indirect markers of muscle damage [5,6]. When evaluating the physical demands of tennis, this acute muscular response should be considered in the context of the competitive season.

The National Collegiate Athletic Association (NCAA) tennis pre-season (including both singles and doubles tournaments) typically runs from September/October to November/December, while the official NCAA tennis season runs from January to May [4]. The NCAA has a 20 h rule which states that countable athletically related activities may occur no more than 20 h per week, with a maximum of 4 hours per day [7]. Despite that rule, the NCAA counts all competition (and any associated activity on the day of competition that might include pre- and post-match practice) as three hours regardless of the duration of these activities [7]. With dual tennis matches during the official NCAA season and tennis tournaments during the preseason having a potential to last longer than 3 h, tennis athletes end up spending a significant amount of time on the tennis court. Since merely playing 2 h of tennis per day has been reported to increase indirect markers of muscle damage and DOMS [5], it is reasonable to assume that training for 20+ hours per week on and off the tennis court might lead to high levels of muscular stress which could result in an increased number of injuries. 

Emerging literature suggest that strength and conditioning (S&C) programs may play an important role in injury prevention within soccer [8,9]. However, the majority of the literature on tennis focuses on S&C practices and performance (as opposed to injury prevention). For example, Kraemer et al. [10] examined the influence of resistance training volume and periodization on physiological and performance variables in collegiate women tennis players. Following the intervention, there were significant increases in fat-free mass, decreases in body fat %, increases in power output, increased maximal strength, and increased serve velocity when performing periodized resistance training alongside tennis training [10]. Based on these data, authors suggested that periodized, multiple-set resistance training programs are most effective for long-term performance increases in collegiate athletes [10]. It appears that S&C programs may enhance performance; however, their impact on injury prevention is largely unknown. 

Although there is a lack of literature exploring the impact of S&C programs on injury rates in tennis, there is emerging literature in the sport of soccer. For example, Askling et al. [8] examined the ability of S&C practices to curtail hamstring injuries in elite male soccer players. The authors recruited 30 male soccer players from 2 soccer leagues in Sweden. The athletes were divided into control and training groups where both groups followed the same training protocol with the exception of the intervention group which implemented additional specific hamstring training days [8]. Throughout the duration of the study (10 months), 3/15 injuries occurred in the intervention group (*p* < 0.05) compared with 10/15 injuries in a control group [8], suggesting that injury prevention may be facilitated through the addition of heavy eccentric work to muscle groups typically more prone to injury [8]. Furthermore, Peterson et al. [9] examined the preventative effect of eccentric training on hamstring injuries in soccer players. The authors recruited 461 soccer players to be a part of their intervention group and 481 players to be a part of their control group. Both groups followed their normal training routine with the exception of an intervention group that implemented a Nordic hamstring exercise in addition to their training routine [9]. In total, 27 sessions of the Nordic hamstring exercise were performed in a 10-week period during the midseason break [9]. Following the study, a total of 67 acute hamstring injuries were reported: 15 injuries in the intervention group and 52 injuries in the control group [9]. Together, these studies highlight the potential for S&C practices to play a role in reducing injury in sport. 

Little is known about the relationship between S&C practices and injuries in the sport of tennis. Since identification of the most common tennis injuries is crucial in order to design a S&C training program that will focus on injury prevention [11], the aim of this study was to examine the relationship between the injury rates and S&C practices in the sport of tennis. We hypothesized that there will be a significant correlation between injury rates amongst tennis players and the incorporation of eccentric exercises. Additionally, we wanted to develop a better understanding of S&C practices in collegiate tennis and what aspects of training the teams tend to focus on.

## 2. Materials and Methods

### 2.1. Participants

Over a period of 3 months (from 25 August 2020 to 2 November 2020), 1129 emails were sent out to both men’s and women’s head and/or assistant tennis coaches in NCAA Division 1, 2, and 3 colleges as well as National Association of Intercollegiate Athletics (NAIA) colleges. Coaches were invited to participate in the survey-based study examining injuries and S&C practices in tennis. The response rate was 9.8% resulting in 111 teams completing the survey. 

### 2.2. Design and Procedures

This study was reviewed by the University’s Institutional Review Board and determined to be “exempt” given the survey-based nature of the data collection. Inclusion criteria included coaches, assistant coaches, S&C coaches and athletic trainers of divisions 1,2 and 3 tennis with knowledge of team injuries and S&C practices. Emails of collegiate tennis coaches were collected from university websites through a google search. Participants for this study were recruited through emails and text messages to head and assistant coaches, word of mouth, and a newsletter from the Intercollegiate Tennis Association (ITA). The ITA sent out the link to the survey in their Newsletter to collegiate tennis coaches. Additionally, 1129 emails were sent out by our research team to both men’s and women’s head and/or assistant tennis coaches in NCAA Division 1, 2, and 3 colleges as well as NAIA colleges, inviting them to participate in the survey. In two instances, the tennis coach did not possess the adequate knowledge to complete the survey; thus, the survey was completed by the S&C coach or an athletic trainer. The emails that were sent out to the coaches contained a link to the survey. Once participants clicked on the link in the email, they were asked to consent to complete the survey. After consenting to complete the survey, coaches were able to access the survey immediately. The informed consent and the survey took about 10 min to complete. Participants had twelve weeks to complete the survey before the survey closed. 

### 2.3. Survey

This study was a self-reported online survey questionnaire that was designed through Qualtrics. After logging into the survey, participants were asked to identify their role with the tennis team (assistant or head coach, athletic trainer, strength coach), what teams they worked with (men’s tennis, women’s tennis, or both), and what division of tennis they worked/coached in (Division I, II, III, or NAIA). Given this was a pilot study to learn more about S&C practices in collegiate tennis, the survey utilized has not been validated. However, our injury data show similar trends to other studies [4]. No other studies, to our knowledge have examined specific S&C practices. Thus, our S&C data serve as a starting point to glean insight and develop future studies in this area. 

### 2.4. Injury Identification

Separately, for both men’s and women’s tennis teams, coaches were asked to identify how many of the injuries their team experienced within the last year (including pre-season and competition season). The individuals were able to move the dial to the right to indicate up to 100 injuries for a particular injury. The list of provided injuries included: ankle sprain, ankle fracture, paraspinal muscle strain (lower back), rib muscle strain, lumbar disc degeneration and herniation, abdominal muscle strain, thigh muscle strain, knee ligament sprain/rupture, extensor tendinopathy/rupture, groin muscle strain, extensor carpi ulnaris tendinitis/subluxation (pinky side of the hand), carpal ligament sprain (wrist), lateral elbow tendinopathy, medial elbow tendinopathy, rotator cuff tendinopathy/tear, superior labrum anterior-to-posterior (SLAP) tears, and internal or subacromial impingement (shoulder). The list of injuries was based on the information from Lynall et al. [4], in addition to discussions with tennis coaches and athletes. 

### 2.5. Strength and Conditioning Practices

Coaches were asked to identify the training focus of their tennis team. Specifically, coaches were asked to indicate “yes” or “no” as to whether their team focuses on training for: maximal upper-body strength, upper-body muscle growth, upper-body power, maximal eccentrics in the upper body, maximal lower-body strength, lower-body muscle growth, lower-body power, flexibility and mobility, sports specific training, plyometric training, and wrist and forearm strength. Additionally, they were asked to rank the following characteristics of S&C by level of importance with 1 being the most important and 8 being the least important: maximal muscle strength, maximal muscle power, injury prevention exercise, flexibility and mobility training, muscle growth, sports specific training, bodyweight exercises, and core exercises. 

Finally, coaches were asked to indicate (answer yes or no) whether the following exercises were included in the tennis S&C program: bench press, deadlift, squat, RDL, Olympic lifts, Nordic hamstring curl, overhead press, biceps curls, triceps extensions, leg press, box jumps, military press, upper-body band work, lower-body band work, rotator cuff exercises, planks, lat pulldowns, and pull ups. These exercises were included in the survey based on the training program for collegiate tennis players from the Kraemer et al. [10] study. In addition, exercises were chosen based on conversations had between the research team and tennis coaches about what they considered to be important exercises for their tennis teams.

### 2.6. Analysis

All data were analyzed using SPSS 26.0 (SPSS Inc., Chicago, IL, USA). Descriptive statistics are provided for injuries and S&C practices. In order to examine the relationship between S&C practices and injury rates, separate multiple linear regression analyses were performed for the upper body and lower body using the enter method in SPSS. In the upper body, total upper-body injuries were used as the dependent variable, with training for upper-body strength (yes vs. no) training for upper-body hypertrophy (yes vs. no), training for upper-body power (yes vs. no) and training upper-body eccentric focused work (yes vs. no) used as predictor variables. In the lower-body, total lower-body injuries were used as the dependent variable, with training for lower-body strength (yes vs. no) training for lower-body hypertrophy (yes vs. no), training for lower-body power (yes vs. no) and training with lower-body eccentric focused work (yes vs. no) used as predictor variables. Statistical significance was set at *p* < 0.05. 

## 3. Results

**Figure 1:**
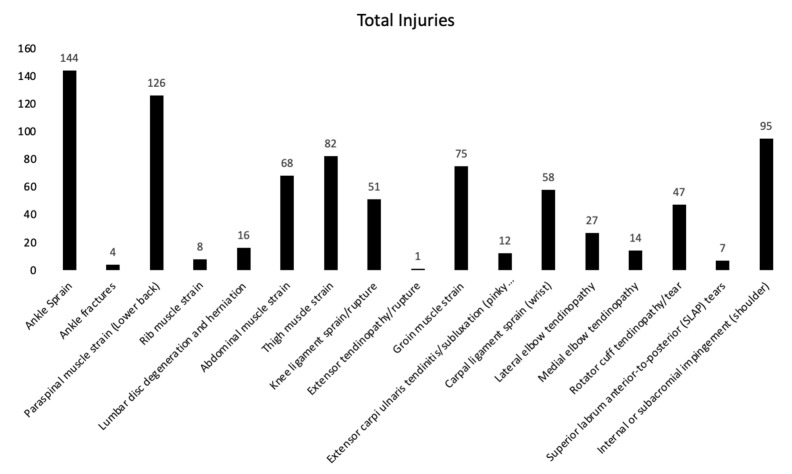
Total number of injuries for all surveyed injuries.

A total of 111 teams’ coaches were surveyed. The number of teams and respondents are presented in Table 1. Total injuries are reported in Figure 1. The most frequent injuries observed were ankle sprains (144 injures), followed by paraspinal muscle strains (126 injuries). In addition, there were 95 internal or subacromial impingements, 82 thigh muscle strains, 75 groin muscle strains, and 68 abdominal muscle strains. When pooled, there were a total of 355 lower-body injuries and 260 upper-body injuries reported. 

**Figure 2:**
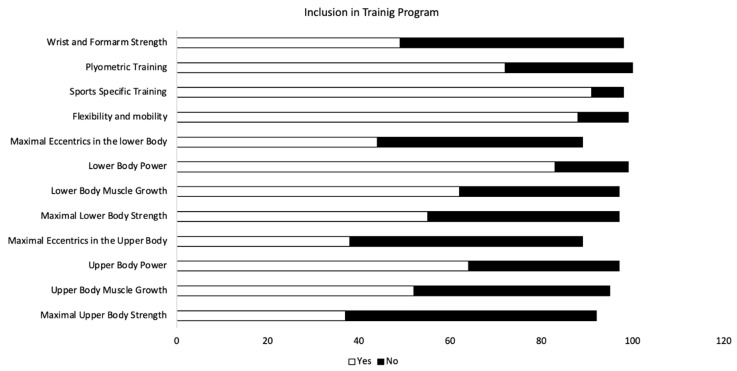
Results for aspects of strength and conditioning. Number of respondents answering “yes” versus number of respondents answering “no” are displayed for each aspect of strength and conditioning.

**Figure 3:**
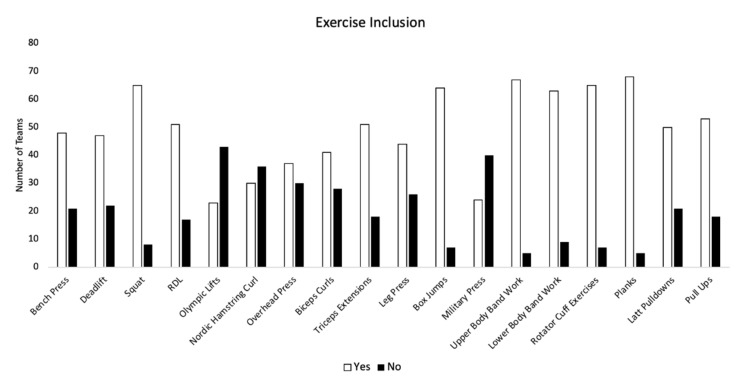
Results for specific exercises included in the S&C program. Number of respondents answering “yes” versus number of respondents answering “no” are displayed for each exercise.

**Figure 4:**
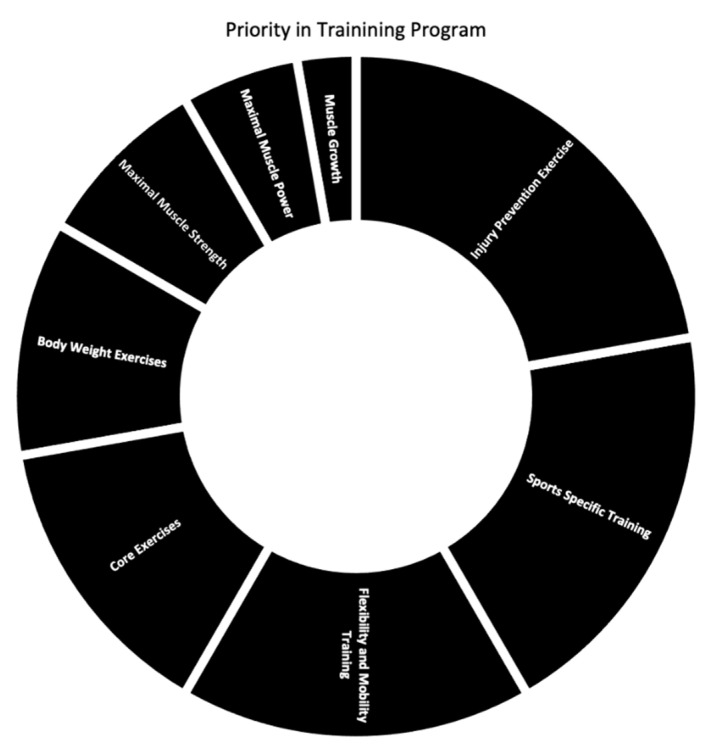
Pie chart representing the rank of various aspects of S&C within the tennis program. Based on results, we ranked aspects 1–8. Accordingly, the largest section (injury prevention exercise) was ranked as most important and the smallest section (muscle growth) was ranked as least important.

When examining S&C practices, 92% of teams reported that their S&C programs included “sports specific training” and 88% of teams indicated that they incorporate flexibility and mobility training. In total, 83% of teams indicated that they incorporate lower-body power and 72% of teams indicated that they incorporate plyometric training. Conversely, only 40% and 42% of teams indicated an incorporation of maximal upper-body strength training and maximal eccentric training in the upper body, respectively. Responses to all aspects of S&C are displayed in Figure 2. Responses to specific exercises within the S&C program are provided in Figure 3. In addition, Figure 4 displays the ranking of aspects of S&C according to the coaches. 

Although aspects of S&C were ranked 1–8 (1 = most important, 8 = least important), in the pie chart, this is reversed so the most important aspect was assigned a value of 8 and the least important aspect was assigned a value of 1. When examining the relationship between S&C practices and injuries, regression analysis revealed that S&C practices explained 9.9% of the variance of injury rates in the upper body (R^2^ = 0.099). The only significant predictor of upper-body injury was participation in training related to upper-body muscle growth (β = 1.613, *p* = 0.013, Table 2). In addition, S&C practices explained 11.1% of the variance of injury rates in the lower body (R^2^ = 0.111). The only significant predictor of lower-body injury was participation in training related to lower-body muscle growth (β = 1.687, *p* = 0.038, Table 3).

## 4. Discussion

The present manuscript is the first (to our knowledge) to examine the relationship between S&C practice and injuries in the sport of tennis. Our primary findings were that the most frequent injuries observed were ankle sprains (144 injures), followed by paraspinal muscle strains (126 injuries), internal or subacromial impingements (95 injuries), and thigh muscle strains (82 injuries). In addition, we found that coaches valued sports-specific training, as well as flexibility and mobility training. Finally, the incorporation of hypertrophy-oriented training appeared to explain a small percentage of the variance in injury rates in both the upper and lower-body. However, S&C practices were overall not strongly predictive of injuries in tennis. 

### 4.1. Injuries

Our results on injury rates are in line with previous studies. For example, Lynall et al. [4] reported that ankle sprains are the most prevalent injuries in the lower-body in both men’s and women’s tennis, followed by trunk injuries, and, lastly, shoulder/clavicle injuries in the upper body. We observed a similar pattern as Lynall et al. [4], with ankle sprains being our top injury followed by lower back strains and shoulder injuries. Kibler and Safran [2] identified thigh and ankle injuries as the most prevalent injuries in the lower-body, followed by elbow and shoulder injuries in the upper-body and back injuries in the central part of the body. Similar rates of injuries across the literature have demonstrated that the prevalence of injuries among tennis players in the past 10 years have not significantly changed. It could partially be attributed to the nature of on-court movement and tennis technique which has remained consistent throughout the years. For example, it has been suggested that ankle sprains are a result of the running, pivoting, stopping, starting, lunging, and jumping movements, which result in high twisting forces that lead to lateral sprains [2]. Additionally, it has been suggested that explosive plantar flexion and internal rotation on an inverted ankle [12] could also result in ankle sprains.

The high prevalence of lower back injuries in tennis may be explained by a smaller range of lower back flexibility in tennis players compared to other athletes. Chandler et al. [13] obtained flexibility measurements from 86 junior elite tennis players and compared them to 139 athletes involved in a variety of other sports. Lower back flexibility was measured in the sit and reach a position in centimeters. Interestingly, tennis players had a back flexibility of only 2.3 cm compared to 6.2 cm of other athletes (*p* < 0.05). Additionally, Chandler et al. [13] observed a non-significant trend toward lower hamstring flexibility in tennis players compared to other athletes which was explained by Kovacs as “the need for tennis players to be in a typical low ready position” [14]. A possible indirect involvement of tight hamstrings and lower back pain has been previously reported in the literature [15,16], which could potentially explain the prevalence of lower back injuries among tennis players. Furthermore, tennis results in a significant asymmetry in both upper and lower-body [17] and, interestingly, postural asymmetry has been associated with compensatory movement of the lumbar spine which can result in increased lower back stress and injury [16]. 

The third most prevalent type of injuries were internal or subacromial impingements of the shoulder, which are the most common causes of shoulder pain amongst overhead athletes [18]. Several mechanisms have been suggested as causes of internal impingements that include excessive humeral translations and tightness of the posterior structures [18]. Excessive humeral translation is typically caused by shoulder instability which could result from shoulder overuse during serves and overheads [18]. Additionally, overhead athletes tend to experience “reduced internal rotation range of motion (ROM) of the dominant arm” which can limit internal joint rotation and contribute to injuries [18]. Finally, it has been suggested that the kinetic chain links upper and lower extremity through transmission of coordinated motion that starts in the legs and moves to the upper body [19]. Thus, any processes that disturbs lower-body, groin, hip and abdominal musculature could increase risk of injuries in the upper body [19].

### 4.2. Injures and Strength and Conditioning Practices 

Our regression analysis suggested that the inclusion of lower-body muscle growth exercises may increase the number of injuries (*p* = 0.038). The narrative was similar in the upper body with the only significant predictor for injuries being the inclusion of training for muscle growth (*p* = 0.013). Dankel et al. [20] indicated that “the level of fatigue caused by an exercise protocol is a good indicator if its hypertrophic potential” suggesting that protocols designed to train for growth typically induce higher levels of fatigue. Muscle fatigue can lead to progressive loss of strength and muscle-tendon stiffness [21], accumulation of metabolic by-products [22], and it has been associated with impaired injury protection mechanisms [22]. Thus, it is possible that tennis teams that trained for muscle growth may have experienced higher levels of fatigue. It is reasonable to suggest that, in some cases, enough time to recover between practices and matches may help explain this relationship. Buckner et al. [23] hypothesized that S&C practice may add unnecessary stress on the body and stated that “resistance training may ultimately take time and recovery away from the performance of the actual sport” suggesting that athletes might consider spending more time practicing their sport and less time performing resistance training protocols. This concept may gain additional support through recent interpretations of Hans Selye’s general adaptation syndrome. Specifically, Buckner et al. [24] have suggested that the general adaptation syndrome may imply that stress management is of greater concern when resistance training is combined with sport [24]. Accordingly, it is important to account for the athletes stress from life, sport and resistance training as all these factors may influence their ability to adapt [24]. However, it is important to note that the applications of the general adaptation syndrome and its underlying theory (adaptation energy) have been questioned in a human model [24,25]. 

### 4.3. Strength and Conditioning Practices 

When examining S&C practices, sport-specific training seemed to be included in the training program by the majority of the tennis coaches, with flexibility and mobility, and lower-body power also commonly included in the majority of programs. These focuses seem reasonable given the demands of tennis. Reid and Schneiker [26] suggested that an ideal in-season tennis S&C program may focus on “developing strength and power without concomitant hypertrophy, increase local muscular endurance, prevent detraining and potentially reduce the risk of injury”. This is in line with the suggestion of Buckner et al. [23] to de-emphasize hypertrophy and focus on sport-specific needs and injury prevention within a S&C program. This suggestion is based on a lack of experimental evidence that increasing muscle size can have a meaningful impact on strength or sports performance [23,27]. In addition, the results of the present study found that eccentric training in the upper body, maximal upper body strength and eccentric training in the lower body were included in the training program by the least amount of tennis teams. This finding may warrant further investigation given the potential of eccentric exercise to decreased injuries rates [8,9]. 

When examining how coaches ranked different aspects of a S&C program by the level of importance, injury prevention exercises were found to be the most important aspect with an average score of 2.27 (1 = most important and 8 = least important), followed by sports specific training with the score of 3.01. Given the prevalence of injury in tennis [4] the high ranking of injury prevention exercises is not surprising. Sport-specific training has been shown to improve aerobic performance similarly to mixed training (combination of HIIT and sport-specific drill training) [28]. Thus, the inclusion of sports specific training likely provides direct and indirect benefits, which appears recognized by coaches. Flexibility and mobility training was ranked third, with an average score of 3.04, followed by core exercises with an average score of 3.86 and body weight exercises with an average score of 5.3. Finally, the three least important aspects of the S&C program included maximal muscle strength with the score of 5.96, followed by the maximal muscle power at 6.05, and muscle growth at 6.5. Interestingly, muscle growth was ranked as the least important aspect of a S&C program. Although the findings of the present study regarding injuries are not conclusive, given the additional volume and muscular stress necessary for hypertrophy training, this may be beneficial for stress management. 

Interestingly, when examining specific exercises, squats, upper and lower-body band work, planks, box jumps, and rotator cuff exercises were included by majority of the coaches. Both band work and rotator cuff exercises could be classified as a form of sport-specific training. Given that sport-specific training was ranked as the second most important aspect of S&C programs, it is not surprising that abovementioned exercises were utilized by majority of tennis coaches. Furthermore, core exercises were ranked as the third most important aspect of the S&C program and not surprisingly, planks were utilized by majority of the coaches. Olympic lifts were utilized by the least number of coaches which could be explained by a complicated nature of the Olympic lifts. Additionally, Nordic hamstring curls were not favored by the coaches which was surprising since, based on the previous soccer data [8,9], there is evidence that addition of eccentric exercises to the S&C program might contribute to injury prevention in the hamstring region. This warrants further investigation into the relationship between eccentric exercises and injuries in tennis players. 

This study is not without limitations. While Lynall et al. [4] derived their data from the NCAA ISP (prospective surveillance program) and Kibler and Safran [2] acquired their information from published sources, our data were obtained from collegiate tennis coaches. Since our survey was not validated, it is possible that not all coaches responded accurately due to their lack of knowledge on injuries or S&C practices. Nonetheless, rates of injuries were similar to Lynall et al. [4] and Kibler and Safran [2]. In addition, coaches were made aware that the survey would inquire about S&C practices and that they are being recruited because of their knowledge of both injury rates and S&C practices. Despite this, there was no question regarding their role/expertise in each of these domains. In addition, our survey was limited by the choices provided. There may have been injuries not captured by the present survey or S&C practices and exercises not included in the present survey. However, the scientific literature was utilized along with expert opinion from coaches in order to create the survey utilized. We also did not account for different periodization strategies or phases of training. However, different aspects trained during different phases should be represented since questions were phrased in the context “over the past year”. Lastly, we quantified the number of injuries and did not examine the severity or duration of each injury which may also be an important consideration. However, no other studies, to our knowledge, have examined specific S&C practices. Thus, our S&C data serve as a starting point to glean insight and develop new further studies in this area. Future research is needed to better understand strategies to reduce injuries in tennis and to better understand the relationship between specific S&C exercises and various injuries in tennis. 

## 5. Conclusions

Results of the present study suggest that coaches value injury prevention exercise, sports specific training and flexibility and mobility training the most, with muscle growth and maximal power ranked lowest. Additionally, the most frequent injuries observed in collegiate tennis players were ankle sprains (144 injures), followed by paraspinal muscle strains (126 injuries), internal or subacromial impingements (95 injuries), and thigh muscle strains (82 injuries).

## Figures and Tables

**Table 1:** Characteristics of Respondents.

	Number of Teams or Respondents (%)
Teams Surveyed	
Total	111
Division I	38 (34%)
Division II	12 (11%)
Division III	58 (52%)
NAIA	3 (3%)
Teams by Sex	
Women’s Tennis Teams	71 (64)
Men’s Tennis Teams	40 (36)
Respondents	
Head or assistant Coaches	109 (98)
Athletic Trainer	1 (<1%)
Strength and Conditioning Coach	1 (<1%)

**Table 2:** Regression Analysis Injuries in Upper Body.

	Model	Unstandardized Coefficients		Standardized Coefficients	Sig.
		β	Std. Error	β	
1	(Constant)	1.062	0.502		0.038
	Strength	1.267	0.688	0.244	0.069
	Growth	1.613	0.632	0.316	0.013
	Power	−0.049	0.652	−0.009	0.94
	Eccentric	−0.861	0.665	−0.167	0.199

Dependent Variable: Upper-body Injuries Total

**Table 3:** Regression Analysis Injuries in Lower body.

	Model	Unstandardized Coefficients		Standardized Coefficients	Sig.
		β	Std. Error	β	
1	(Constant)	1.321	0.83		0.115
	Strength	1.313	0.851	0.194	0.127
	Growth	1.687	0.8	0.243	0.038
	Power	0.499	1.13	0.057	0.66
	Eccentric	−0.829	0.824	−0.124	0.317

Dependent Variable: Lower-body Injuries Total

## Data Availability

Data available upon request.
